# Stress Granule Core Protein-Derived Peptides Inhibit Assembly of Stress Granules and Improve Sorafenib Sensitivity in Cancer Cells

**DOI:** 10.3390/molecules29092134

**Published:** 2024-05-04

**Authors:** Juan Li, Yaobin Zhang, Jinxuan Gu, Yulin Zhou, Jie Liu, Haiyan Cui, Tiejun Zhao, Zhigang Jin

**Affiliations:** 1 College of Life Sciences, Office of Student Entrepreneurship, Zhejiang Normal University, 688 Yingbin Road, Jinhua 321004, China; 2 Key Laboratory of Novel Targets and Drug Study for Neural Repair of Zhejiang Province, School of Medicine, Hangzhou City University, Hangzhou 310015, China

**Keywords:** stress granules, G3BP1, USP10, Caprin1, anticancer drug resistance, sorafenib

## Abstract

Upon a variety of environmental stresses, eukaryotic cells usually recruit translational stalled mRNAs and RNA-binding proteins to form cytoplasmic condensates known as stress granules (SGs), which minimize stress-induced damage and promote stress adaptation and cell survival. SGs are hijacked by cancer cells to promote cell survival and are consequently involved in the development of anticancer drug resistance. However, the design and application of chemical compounds targeting SGs to improve anticancer drug efficacy have rarely been studied. Here, we developed two types of SG inhibitory peptides (SIPs) derived from SG core proteins Caprin1 and USP10 and fused with cell-penetrating peptides to generate TAT-SIP-C1/2 and SIP-U1-Antp, respectively. We obtained 11 SG-inducing anticancer compounds from cell-based screens and explored the potential application of SIPs in overcoming resistance to the SG-inducing anticancer drug sorafenib. We found that SIPs increased the sensitivity of HeLa cells to sorafenib via the disruption of SGs. Therefore, anticancer drugs which are competent to induce SGs could be combined with SIPs to sensitize cancer cells, which might provide a novel therapeutic strategy to alleviate anticancer drug resistance.

## 1. Introduction

Cells are inevitably exposed to a variety of adverse conditions such as oxidative stress, osmotic stress, heat shock, nutrient deprivation, viral infection, hypoxia, and pharmacological treatment. These stresses could derail cellular homeostasis and impose a threat to cell survival. Eukaryotic cells have evolved multiple defensive strategies to facilitate stress adaptation and cell survival and restore cellular homeostasis. For example, as a way of crisis management, cells usually block the global translation of mRNAs to reduce energy consumption while selectively stimulating the translation of mRNAs that support stress adaptation and cell survival. Meanwhile, these untranslated mRNAs and translation initiation factors should be kept from damage or degradation so that translation could be quickly re-initiated after recovery from stress. To achieve this, cells often elicit the assembly of stress granules (SGs), a type of cytoplasmic membraneless condensates that sequester translationally inactive mRNAs, translation initiation factors, RNA-binding proteins, and signaling proteins for transient storage [1,2,3,4]. The elicitation of SGs is an important and conserved cellular strategy that regulates protein translation and cell signaling, thereby minimizing stress-related damage and promoting cell survival. Stress-induced SGs are usually transient and reversible and are disassembled once stress is adapted or no longer exists [5,6].

SGs could be induced via two pathways that mediate the stress-induced suppression of translation initiation. The first one is the phosphorylation of the α subunit of eukaryotic initiation factor 2 (eIF2α) at serine 51, which limits the availability of the eIF2–GTP–Met-tRNA_i_^Met^ ternary complex required for translation initiation, leading to the suppression of global translation and activation of gene-specific translation such as activating transcription factor 4 (ATF4) and C/EBP homologous protein (CHOP). The second one is the disruption of eIF4F, the cap-binding complex that is composed of eIF4A, eIF4E, and eIF4G, and drives translation initiation via recruiting ribosomes to the 5′ end of mRNAs [7,8]. Accordingly, SGs could be classified as eIF2α phosphorylation-dependent SGs and independent SGs. During the induction of eIF2α phosphorylation-dependent SGs, four types of stress-activated kinases are responsible for the phosphorylation of eIF2α. General control nonderepressible 2 (GCN2) is activated by the amino acid depletion-induced accumulation of uncharged tRNAs. The heme-regulated inhibitor (HRI) is activated by oxidative stress and heme deficiency in erythroid cells. The double-stranded RNA-dependent protein kinase (PKR) is activated by viral infections. The PKR-like endoplasmic reticulum kinase (PERK) is activated by ER stress. On the other hand, during the induction of eIF2α phosphorylation-independent SGs, the assembly of the eIF4F complex was interrupted, mainly by the inhibition of RNA helicase eIF4A or a blocking interaction between the cap-binding protein eIF4E and scaffold protein eIF4G [1,2,3,4]. 

The assembly of SGs is a complicated process that involves liquid–liquid phase separation (LLPS) arising from multivalent protein–protein, protein–RNA and RNA–RNA interactions. Recent studies have revealed that around 36 SG core proteins constitute a core SG network, within which Ras-GTPase-activating protein SH3 domain-binding protein 1 and 2 (G3BP1/2) function as the central node [9,10,11]. G3BP1/2 double knockout cells fail to assemble SGs, including both eIF2α phosphorylation-dependent and independent SGs. Not surprisingly, G3BP1/2 undergo tight regulation during the assembly and disassembly of SGs. Cell cycle-associated protein 1 (Caprin1) and ubiquitin-specific peptidase 10 (USP10) are among the 36 SG core proteins and are also of great significance in the regulation of G3BP1/2 by competitive binding to the N-terminal NTF2-like (NTF2L) domain of G3BP1/2 with opposite outcomes. The interaction of G3BP1/2 with Caprin1 promotes SG assembly, while interaction with USP10 inhibits SG assembly [6,9,12,13,14].

The major function of SGs is to cope with stress and ensure cell survival. The dysregulation of SG dynamics such as the inhibition of SGs or transition to aberrant SGs is implicated in the pathogenesis of many human diseases including cancer, neurodegenerative diseases, and viral infections. Upon viral infections, host eukaryotic cells usually form SGs to restrict viral replication via the repression of viral translation. Viruses have evolved various strategies to inhibit the assembly of SGs, one of which is to target G3BP1/2 by viral proteins containing the FGDF motif, resulting in SG inhibition or remodeling to pro-viral condensates [13,15]. We have recently found that a similar strategy was employed by the SARS-CoV2 nucleocapsid protein to remodel SGs and facilitate viral infection [16,17]. Interestingly, the N-terminus of USP10 also contains an FGDF motif, which confers the capacity of SG inhibition to USP10 [6,12,13,14]. 

Growing evidence shows that SGs have emerged as a key player in tumor progression by integrating oncogenic signaling and tumor microenvironment-associated stress to inhibit the apoptosis of cancer cells and promote the proliferation, invasion, and migration of cancer cells [1,18,19,20,21]. Oncogenic signaling, including the PI3K/mTOR pathway and cancer-associated KRAS mutant, markedly elevates SG formation to enhance cancer cell fitness [19,22,23]. Cells with defects in SG assembly undergo massive stress-induced apoptosis, supporting the anti-apoptotic and stress-adaptive role of SGs [24]. However, this property of SGs has been hijacked by cancer cells to facilitate stress adaptation and cell survival under tumor microenvironment-associated stress, such as hypoxia, oxidative stress, ER stress, hyperosmolarity, and nutrient deprivation [1,20,25]. Furthermore, SGs also mediate the development of cancer treatment resistance, including resistance to chemotherapy, radiotherapy, and targeted therapy. Various anticancer drugs such as sorafenib, lapatinib, 5-fluorouracil (5-FU), bortezomib, docetaxel, and vinorelbine could induce eIF2α phosphorylation-dependent SGs that exert a pro-survival effect and contribute to drug resistance and compromised therapeutic outcomes [1,26,27,28,29,30,31,32]. The inhibition of SGs by targeting eIF2α kinases HRI or PERK could improve the sensitivity of cancer cells to SG-inducing anticancer drugs [26,29,32], indicating that SG inhibitors could be employed as a therapeutic strategy to promote the efficacy of cancer treatment and alleviate anticancer drug resistance. It also raises the significance of the development of SG inhibitors. Although efforts have been paid to screen SG inhibitors from compound library screens [33,34,35,36,37], these SG inhibitors are limited by specificity or SG-inhibiting efficacy. The design of compounds based on the SG core protein-mediated SG assembly mechanism and viral anti-SG strategies that afford us opportunities to learn are still lacking [38]. 

In this study, we utilized two different strategies to design SG inhibitory peptides (SIPs) that are able to inhibit SGs. Peptides derived from SG core proteins Caprin1 and USP10 were acquired and fused with a cell-penetrating peptide to generate TAT-SIP-C1/2 and SIP-U1-Antp, respectively. We found that these peptides significantly inhibited sorafenib-induced SGs and increased the sensitivity of human cervical cancer HeLa cells to sorafenib, suggesting the potential application of SIPs in improving the therapeutic effect of SG-inducing anticancer drugs.

## 2. Results 

### 2.1. Design Strategy for SG Core Protein-Derived SG Inhibitory Peptides 

**Figure 1:**
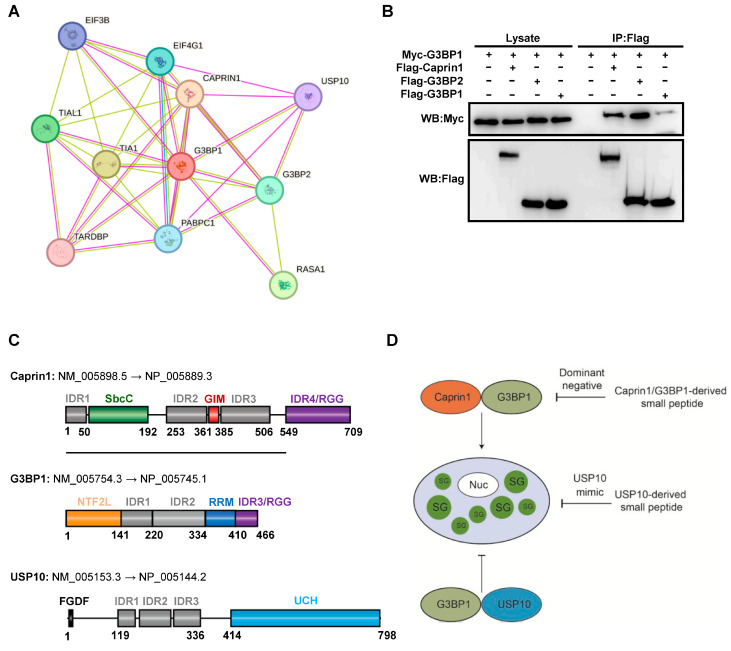
Design strategy for SG core protein-derived SG inhibitory peptides. (**A**) G3BP1 protein–protein interaction network. (**B**) Co-IP showing the G3BP1 and Caprin1 interaction and G3BP1/2 dimerization. HEK293T cells were transfected with indicated expression plasmids and cell lysates were subjected to Co-IP using the anti-Flag antibody, followed by Western blot analysis. (**C**) Schematic representation of functional domains in human Caprin1, G3BP1, and USP10 indicated in different colored boxes. IDR, intrinsically disordered region; GIM, G3BP1-interacting motif; RRM, RNA recognition motif; UCH, Ubiquitin C-terminal hydrolase. (**D**) Schematic representation of design rationale for SIPs derived from SG core proteins Caprin1, G3BP1, and USP10.

The G3BP1 protein–protein interaction network showed that G3BP1 extensively interacts with SG core proteins that contribute significantly to SG assembly [9], such as G3BP2, Caprin1, USP10, and TIA-1 (Figure 1A). We confirmed that G3BP1 physically interacted with Caprin1 and formed the G3BP1–G3BP2 heterodimer and G3BP1–G3BP1 homodimer in unstressed HEK293T cells (Figure 1B). Indeed, a large number of SG proteins interact with G3BP1 in the absence of stress and these pre-existing interactions may facilitate the quick assembly of SGs upon stress [39].

Previous studies showed that two SG core proteins Caprin1 and USP10 play a key role in G3BP1/2-mediated SG assembly. The interaction of G3BP1/2 with Caprin1 promotes SG assembly, whereas USP10 inhibits SG assembly [12,13]. These elegant studies inspired us to utilize two different strategies to design SG core protein-derived SG inhibitory peptides (SIPs) that are able to block the nucleating function of G3BP1, thereby inhibiting SG assembly. The first one is based on the SG-promoting interaction between G3BP1/2 and Caprin1 and we tried to obtain Carpin1 or G3BP1-derived small peptides with a dominant negative effect on the SG-promoting function of G3BP1 (Figure 1C,D). The second one is based on the SG-inhibiting interaction between G3BP1/2 and FGDF motif-containing USP10 or viral proteins and we tried to obtain minimal peptides retaining SG-inhibiting activity.

### 2.2. Identification of Caprin1-Derived SG Inhibitory Peptides

**Figure 2:**
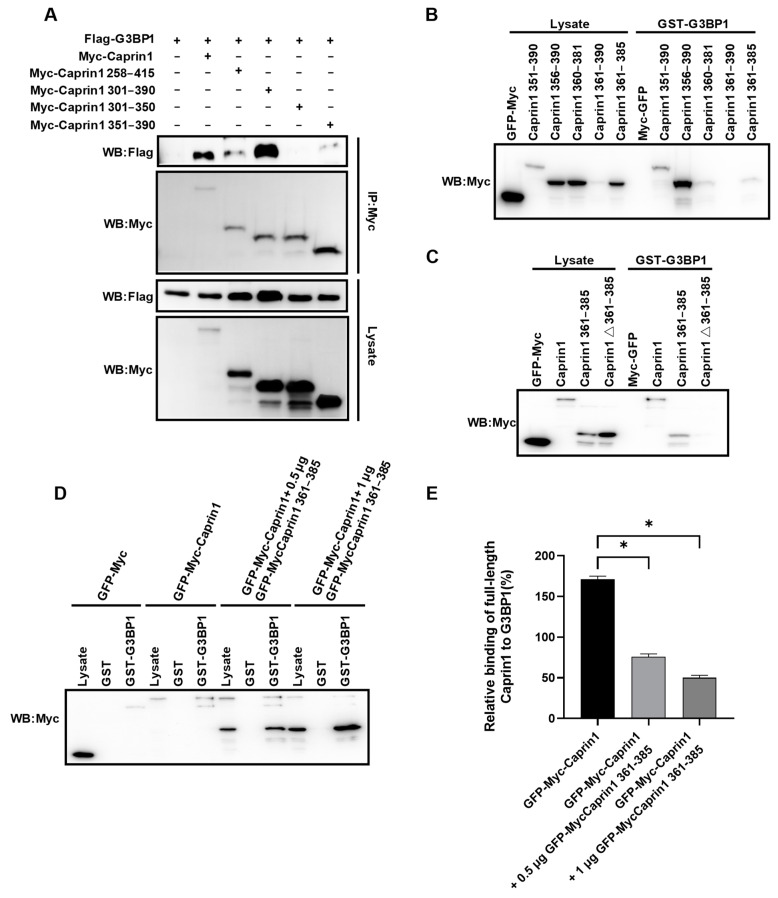
Identification of Caprin1-derived SG inhibitory peptides. (**A**) Co-IP showing the interaction between G3BP1 and serial fragments of Caprin1. HEK293T cells were transfected with indicated expression plasmids and cell lysates were subjected to Co-IP using the anti-Myc antibody, followed by Western blot analysis. (**B,C**) GST pull-down showing the interaction between G3BP1 and serial fragments or internal deletions of Caprin1. HEK293T cells were transfected with indicated expression plasmids and cell lysates were subjected to GST pull-down using GST-G3BP1 beads, followed by Western blot analysis. (**D**) GST pull-down showing that Caprin1 fragment 361–385 competed with full-length Caprin1 to bind G3BP1 in a dose-dependent manner. HEK293T cells were transfected with indicated expression plasmids and cell lysates were subjected to GST pull-down using GST or GST-G3BP1 beads, followed by Western blot analysis. (**E**) Statistical analysis of the binding efficiency of full-length Caprin1 to G3BP1 shown in panel (**D**). * *p* ≤ 0.05.

We then tried to identify small peptides from Caprin1 by serial deletions. Co-IP and GST pull-down assays showed that residues 351 to 390 of Caprin1 retained the ability to interact with G3BP1 (Figure 2A,B). The G3BP1-interacting motif (GIM) could be further narrowed down to residues 361–385; the deletion of this region abolished binding to G3BP1 (Figure 2C). This indicates the sufficiency and necessity of Caprin1 fragment 361–385 to the interaction of Caprin1 with G3BP1, which is consistent with the critical contribution of glycine 368 (G368) and phenylalanine 372 (F372) and the adjacent residues to the interaction [14,40]. To determine if this fragment could compete with the full-length Caprin1 to block the interaction between G3BP1 and Caprin1, we performed the GST pull-down assay with increasing amounts of Caprin1 fragment 361–385. As shown in Figure 2D,E, full-length Caprin1 interacted with GST-G3BP1 with robust affinity. However, the binding of Caprin1 fragment 361–385 to immobilized GST-G3BP1 prevented full-length Caprin1 from binding to GST-G3BP1 in a dose-dependent manner. 

**Figure 3:**
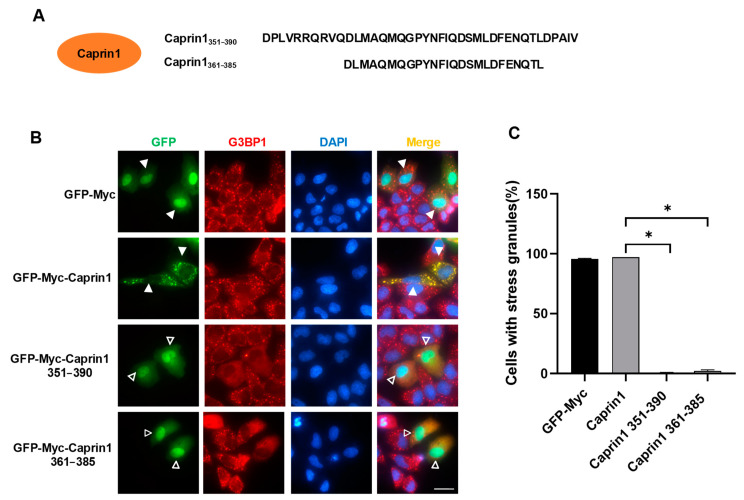
Overexpression of Caprin1-derived fragments inhibits SG assembly. (**A**) Amino acid sequences of Caprin1 351–390 (SIP-C1) and 361–385 (SIP-C2). (**B**) Immunofluorescence showing that overexpression of Caprin1 fragments 351–390 and 361–385 inhibited AS-induced SGs. HeLa cells were transfected with indicated expression plasmids, treated with 0.5 mM AS for 45 min and subjected to immunofluorescence staining using the anti-G3BP1 antibody. Solid triangles indicate cells with SGs while empty triangles indicate cells without SGs. Scale bars: 20 µm. (**C**) Statistical analysis of SG induction efficiency shown in panel (**B**), which is reflected by the percentage of SG-positive cells among cells successfully transfected with expression plasmids. * *p* ≤ 0.05.

As a next step, we investigated the impact of Caprin1 fragments on the assembly of SGs (Figure 3A). G3BP1 was used as a marker of SGs and was induced by sodium arsenite (AS). The overexpression of full-length Caprin1 barely affected SG assembly as compared to the GFP-Myc vector (Figure 3B,C). In contrast, Caprin1 fragments 351–390 or 361–385 almost completely inhibited SG assembly. Taken together, these results showed that Caprin1 fragments 351–390 and 361–385 possess G3BP1-binding ability and are able to block SG assembly via the disruption of G3BP1 and Caprin1 interaction.

### 2.3. Identification of G3BP1-Derived SG Inhibitory Peptides

**Figure 4:**
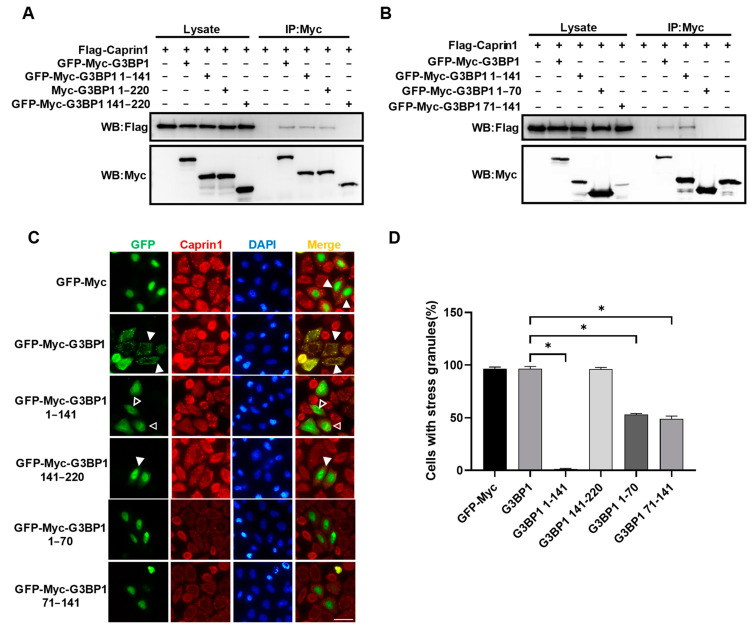
Identification of G3BP1-derived SG inhibitory peptides. (**A,B**) Co-IP showing the interaction between Caprin1 and serial fragments of G3BP1. HEK293T cells were transfected with indicated expression plasmids and cell lysates were subjected to Co-IP using the anti-Myc antibody, followed by Western blot analysis. (**C**) Immunofluorescence showing that only overexpression of G3BP1 fragment 1–141 (NTF2L) effectively inhibited AS-induced SGs. HeLa cells were transfected with indicated expression plasmids, treated with 0.5 mM AS for 45 min and subjected to immunofluorescence staining using the anti-Caprin1 antibody. Solid triangles indicate cells with SGs while empty triangles indicate cells without SGs. Scale bars: 20  µm. (**D**) Statistical analysis of SG induction efficiency shown in panel (**C**), which is reflected by the percentage of SG-positive cells among cells successfully transfected with expression plasmids. * *p* ≤ 0.05.

The N-terminal NTF2L domain of G3BP1 is essential for LLPS of G3BP1 and SG assembly via interaction with Caprin1 and G3BP1 dimerization, and phenylalanine at residue 33 (F33) within NTF2L is critical to interact with Caprin1 [9,12]. Next, we tried to search for G3BP1-derived SIPs within the NTF2L domain that resides in residues 1–141 (Figure 1C). Co-IP assays showed that the Caprin1-interacting motif could be narrowed down to NTF2L (Figure 4A). Surprisingly, two fragments, 1–70 and 71–141, that split from NTF2L were not able to interact with Caprin1 (Figure 4B). We assumed that residues at around position 70 might be important for the interaction but found that G3BP1 fragments 1–100 and 40–141 still failed to bind Caprin1. This indicates that the interaction of G3BP1 NTF2L with Caprin1 depends on both terminal regions of NTF2L. 

We also examined how these G3BP1 fragments affect the assembly of SGs induced by AS. To avoid the disturbance of the transfected G3BP1 protein to SG staining results, we used Caprin1 instead of G3BP1 as the SG marker. We found that full-length G3BP1 or IDR1 (141–220) had little effect on AS-induced SGs, whereas NTF2L inhibited the majority of SGs (Figure 4C,D). However, G3BP1 fragments 1–70 or 71–141 only partially inhibited SGs, with around 50% transfected cells still containing SGs. Overall, the minimal fragment of G3BP1 that binds to Caprin1 and inhibits SGs is NTF2L (1–141). We are not able to obtain G3BP1-derived SIPs with the potential for therapeutic application in regard to peptide length (typically less than 40 aa) [41,42]. 

### 2.4. Identification of FGDF Motif-Containing SG Inhibitory Peptides

**Figure 5:**
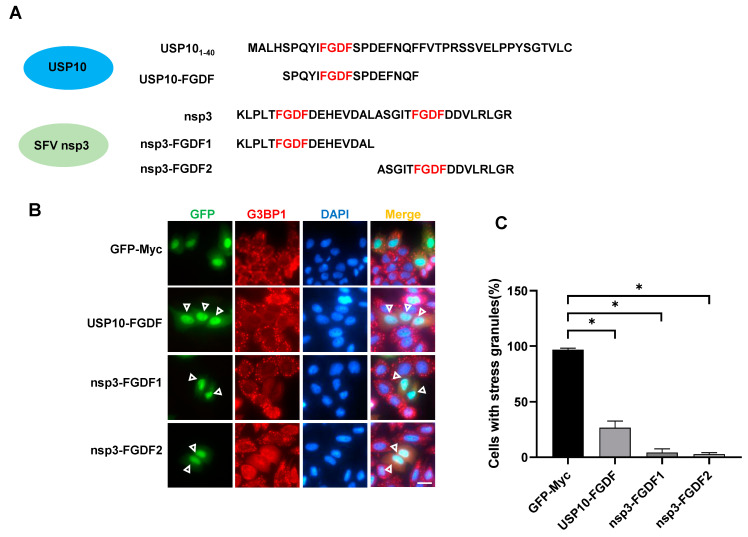
Identification of FGDF motif-containing SG inhibitory peptides derived from USP10 and viral protein nsp3. (**A**) Amino acid sequences of USP10-derived SIP (USP10-FGDF) and nsp3-derived SIPs (nsp3-FGDF1 and nsp3-FGDF2) containing FGDF motif. (**B**) Immunofluorescence showing that overexpression of FGDF motif-containing fragments USP10-FGDF, nsp3-FGDF1, and nsp3-FGDF2 effectively inhibited AS-induced SGs. HeLa cells were transfected with indicated expression plasmids, treated with 0.5 mM AS for 45 min and subjected to immunofluorescence staining using the anti-G3BP1 antibody. Empty triangles indicate cells without SGs. Scale bars: 20 µm. (**C**) Statistical analysis of SG induction efficiency shown in panel (**B**), which is reflected by the percentage of SG-positive cells among cells successfully transfected with expression plasmids. * *p* ≤ 0.05.

USP10 and many viral proteins shared the FGDF motif, which contains FGDF and at least two acidic residues within the five downstream residues and represents a binding model to target G3BP1/2, leading to SG inhibition [12,13]. We then tried to apply the FGDF motif into SIPs and constructed three 17 aa-long fragments. The fragment of USP10 FGDF was derived from USP10 residues 5–21 (Figure 1C and Figure 5A) and two consecutive FGDF motifs from the non-structural protein 3 (nsp3) of the Semliki Forest virus (SFV) were separated as nsp3-FGDF1 and nsp3-FGDF2 (Figure 5A). The immunofluorescence staining results showed that each of the three fragments could effectively block AS-induced SGs (Figure 5B,C), indicating their potential as therapeutic peptides in SG-associated diseases. 

### 2.5. SG Inhibitory Peptides Inhibit Sorafenib-Induced SGs

**Figure 6:**
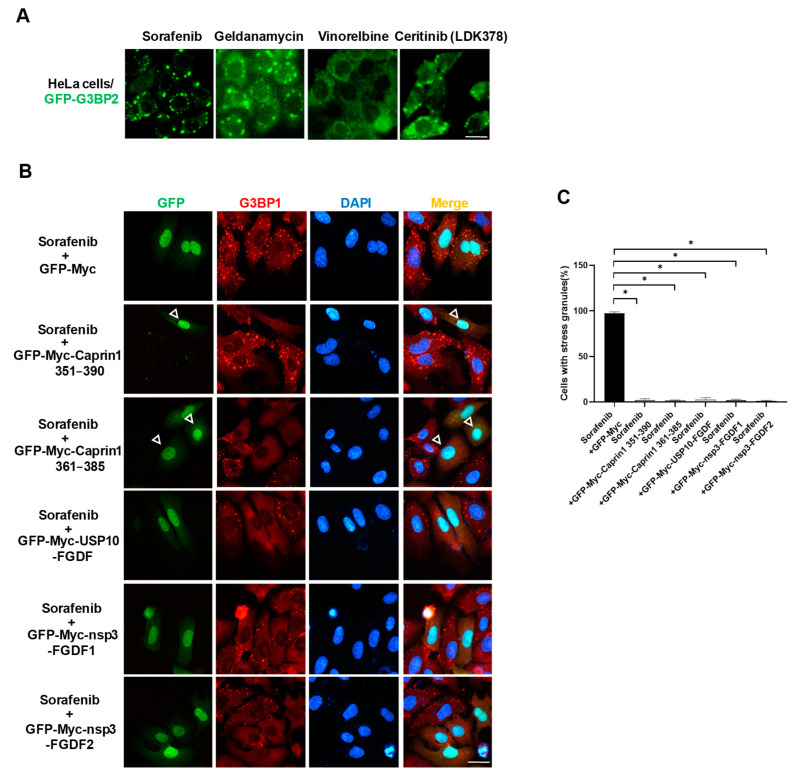
Overexpression of SIP fragments blocks the formation of SGs induced by sorafenib. (**A**) Screen for SG-inducing anticancer compounds. HeLa cells stably expressing GFP-G3BP2 were treated with 10 μM compounds (geldanamycin, vinorelbine, and ceritinib) or 50 μM sorafenib for 2 h and captured for fluorescent images. (**B**) Immunofluorescence showing that overexpression of SIP fragments effectively inhibited sorafenib-induced SGs. HeLa cells were transfected with indicated expression plasmids, treated with 50 μM sorafenib for 2 h and subjected to immunofluorescence staining using the anti-G3BP1 antibody. Empty triangles indicate cells without SGs. Scale bars: 20 µm. (**C**) Statistical analysis of SG induction efficiency shown in panel (**B**), which is reflected by the percentage of SG-positive cells among cells successfully transfected with expression plasmids. * *p* ≤ 0.05.

Previous studies have shown that sorafenib, lapatinib, 5-FU, bortezomib, docetaxel, vinorelbine, and other anticancer drugs could induce eIF2α phosphorylation-dependent SGs, which promote the survival of cancer cells [1,26,27,28,29,30,31,32]. We utilized HeLa/GFP-G3BP2 cells that stably expressed GFP-G3BP2 and carried out a screen from a library pool containing 244 anticancer compounds to retrieve SG-inducing anticancer compounds. In total, we obtained 11 candidates, including geldanamycin, vinorelbine, ceritinib (LDK378), and sorafenib (Figure 6A), among which geldanamycin and ceritinib have never been reported as SG inducers. We found that 10 μM of sorafenib induced SGs with very low efficiency in HeLa cells but 50 μM of sorafenib induced 95% of cells to form SGs. As sorafenib-induced SGs contribute to cancer cell resistance [26,34], we used sorafenib for the following study. 

HeLa cells transfected with expression plasmids containing previously identified fragments were treated with 50 μM of sorafenib. Compared to the GFP-Myc vector, Caprin1 351–390, Caprin1 361–385, USP10-FGDF, nsp3-FGDF1, and nsp3-FGDF2 all significantly suppressed sorafenib-induced SGs in HeLa cells (Figure 6B,C), as well as in HepG2 and SH-SY5Y cells. This indicates that the designed SIPs might be applied to inhibit SGs induced by a wide range of stressors. 

### 2.6. SG Inhibitory Peptides Improve Sorafenib Sensitivity in Cervical Cancer Cells

**Figure 7:**
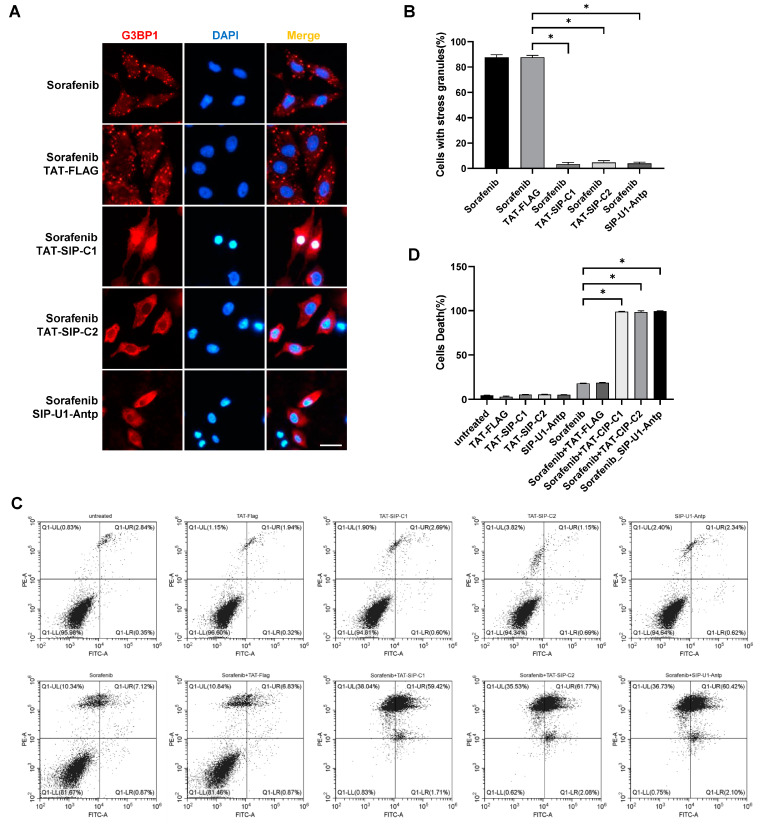
Synthesized peptides TAT-SIP-C1/2 and SIP-U1-Antp inhibit sorafenib-induced SGs and promote the sensitivity to sorafenib in HeLa cells. (**A**) Immunofluorescence showing that TAT-SIP-C1/2 and SIP-U1-Antp significantly reduced sorafenib-induced SGs. HeLa cells were pretreated with peptides for 2 h followed by incubation with 50 μM sorafenib for an additional 2 h, and then subjected to immunofluorescence staining using the anti-G3BP1 antibody. Nuclei of HeLa cells were co-stained with DAPI. Scale bars: 20 µm. (**B**) Statistical analysis of SG induction efficiency shown in panel (**A**), which is reflected by the percentage of SG-positive cells among all cells. (**C**) Annexin V/PI flow cytometry assay showing that TAT-SIP-C1/2 and SIP-U1-Antp significantly increased sorafenib-induced cell death. HeLa cells were pretreated with peptides for 2 h followed by incubation with 50 μM sorafenib for an additional 2 h, and then subjected to Annexin V/PI flow cytometry assay. (**D**) Statistical analysis of cell death shown in panel (**C**), which is reflected by the percentage of dead cells (a combined population of Annexin V^+^PI^+^, Annexin V^+^PI^−^, and Annexin V^−^PI^+^) among all cells. * *p* ≤ 0.05.

Next, we selected three SIPs, Caprin1 351–390, Caprin1 361–385, and USP10-FGDF, to fuse with the cell-penetrating peptide TAT or Antp/Penetratin for delivery across the cell membrane [43,44], and synthesized corresponding peptides, which were named as TAT-SIP-C1, TAT-SIP-C2, and SIP-U1-Antp, respectively. TAT-Flag was also synthesized and served as a control. Then, we pretreated HeLa cells with synthesized peptides, followed by the induction of SGs with sorafenib. Similar to the overexpression assay (Figure 6B,C), the direct treatment of TAT-SIP-C1, TAT-SIP-C2, and SIP-U1-Antp also efficiently blocked the assembly of sorafenib-induced SGs (Figure 7A,B).

Finally, in order to investigate the effect of SG inhibition on drug resistance in cancer cells, we pretreated HeLa cells with peptides followed by sorafenib treatment and performed the Annexin V/PI flow cytometry assay. Our results showed that the treatment of peptides weakly and non-significantly impacted cell death (Figure 7C,D). Cell death caused by sorafenib (18.03%) was barely affected by the combination with TAT-Flag (18.76%), but was markedly increased by the combination with TAT-SIP-C1 (98.91%), TAT-SIP-C2 (98.42%), or SIP-U1-Antp (99.47%). Overall, we concluded that the use of Caprin1 and USP10-derived SIPs is an effective way to promote the efficacy of sorafenib treatment in cancer cells. 

## 3. Discussion

**Figure 8:**
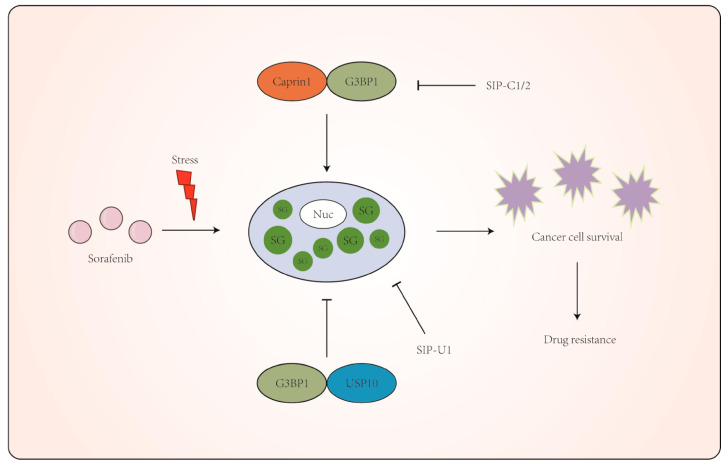
Working model of SIPs that increase the efficacy of sorafenib in cancer cells. Sorafenib imposes stress on cancer cells, which respond to induce SGs that promote stress adaptation and cell survival. SIP-C1/2 and SIP-U1 derived from SG core proteins Caprin1 and USP10 exert a dominant effect and USP10-mimic effect on the SG-promoting function of G3BP1, leading to inhibition on SG assembly and cell survival. Combined treatment of SG-inducing anticancer drugs with SIPs might alleviate SG-associated drug resistance.

The biological composition, function, and dynamic regulation of SGs have attracted significant interest because of the close association between SGs and cancer, neurodegenerative diseases, and viral infections [1,2,3,4]. SGs are an important cellular defense against stress and contribute significantly to stress adaptation and cell survival. However, SGs have been hijacked by cancer cells to minimize apoptosis and promote stress adaptation and cell survival under tumor microenvironment-associated stress. Recently, accumulating evidence has revealed the key role of SGs in promoting the proliferation, invasion, and migration of cancer cells, and the development of cancer cell resistance, which contributes to poor clinical treatment efficacy [1,18,19,20,21]. It is reasonable to assume that compounds specifically targeting SG core proteins to inhibit SG assembly might provide a novel strategy to combat cancer cell resistance. In this study, we designed five small fragments derived from the SG core protein Caprin1, USP10, and FGDF motif-containing viral proteins, and they exhibited a high efficiency for SG inhibition. We further fused Caprin1 and USP10-derived peptides with a cell-penetrating peptide; the synthesized TAT-SIP-C1/2 and SIP-U1-Antp could effectively block sorafenib-induced SGs and improve the sensitivity of cancer cells to sorafenib-induced cell death (Figure 8). 

To what extent SIPs should be narrowed down is a major concern during the design of SIPs. A long peptide would maintain a robust SG-inhibiting capability, but is also inevitably associated with disadvantages, such as non-specificity, side effects, increased delivery difficulty, and synthesis cost. A peptide that is too short, however, might lead to a compromised SG-inhibiting capability. The lengths of our current TAT-SIP-C2 and SIP-U1-Antp are 37 aa (TAT 11 + SIP 26) and 33 aa (SIP 17 + Antp 16); both are within the range of a typical peptide-based drug (less than 40 aa) [41,42]. Whether these peptides can be further narrowed down while maintaining the SG-inhibiting ability, or whether the cell-penetrating peptide can be skipped, owing to small polar surface areas, remain to be further investigated in the future.

As NTF2L of G3BP1 plays a key role in G3BP1–Caprin1 interaction as well as G3BP1/2 dimerization [9], NTF2L-derived SIPs might also be able to suppress SG assembly. We tried to narrow down the NTF2L domain (1–141) of G3BP1 but failed to obtain G3BP1-derived SIPs smaller than NTF2L. Neither of the two NTF2L fragments, 1–70 and 71–141, can bind to Caprin1. Consistently, they also failed to block SG assembly effectively. We speculate that both terminal regions of NTF2L are required for the interaction of G3BP1 with Caprin1 and G3BP1/2 dimerization. If this is the case, it is hard to develop G3BP1 NTF2L-derived SIPs. Nevertheless, the C-terminal RRM and IDR3-mediated interaction of G3BP1 with mRNAs is also essential for LLPS of G3BP1 and SG assembly [9], suggesting that the C-terminal region could also be considered to develop G3BP1-derived SIPs. Indeed, Coxsackievirus type B3 (CVB3) 3C protease cleaves G3BP1 at Q325 and produces a dominant-negative C-terminal fragment that inhibits SG assembly and facilitates viral replication [45]. It is interesting to seek or verify the SG-inhibiting and therapeutic potential of G3BP1-derived SIPs targeting the C-terminal region and other regions outside of NTF2L [46,47]. 

As viruses have evolved multiple strategies to inhibit SGs with an antiviral function [48,49], an easy and smart way is to learn and apply these strategies in SIP design, which is the case for viral protein-derived FGDF motif-containing SIPs. During the preparation of this manuscript, a new study has developed two small molecules, G3BP inhibitor a and b (G3Ia and G3Ib) from nsp3 [38], which have the same origin of our nsp3-FGDF1 and nsp3-FGDF2. G3Ia and G3Ib are only 8 aa in length but are sufficient to bind to a specific pocket in G3BP1/2, disrupt LLPS of G3BP1, and inhibit SG formation across multiple cell types and a variety of stressors. It seems that the development, refinement, and therapeutic application of SIPs will be the intensive focus of future studies. Interestingly, most FGDF motif-containing viral proteins prefer to target the NTF2L domain of G3BP1 with a key role of F33 in this interaction, thereby inhibiting SGs or repurposing G3BP1 for pro-viral function [13,15,16]. In this scenario, the G3BP1 NTF2L-derived peptide encompassing F33 might be able to restore SG assembly and cellular defense by blocking the interaction with viral proteins, indicating its therapeutic potential in viral diseases.

Another interesting finding of our study is that we obtained 11 SG-inducing anticancer compounds from cell-based screens, among which geldanamycin and ceritinib are novel SG inducers. Although we confirmed that a variety of anticancer compounds are able to induce SGs, the relatively low percentage of SG-inducing compounds (11 out of 244) is out of our expectation. Previously reported SG inducers such as 5-FU and docetaxel [28,30] did not induce SGs in HeLa/GFP-G3BP2 cells, but 5-FU induced SGs in human colon cancer HCT116 cells (data not shown). This might be due to the cell type-specificity of SG inducers [39]. This also indicates that different cancer cells should be used to re-test the SG-inducing ability of anticancer compounds. In addition, the outcome of SG induction should also be determined, because SGs induced by a majority of anticancer compounds promote cell survival, while SGs induced by very few anticancer compounds mediate their cytotoxicity, such as hippuristanol and silvestrol [1,2,3,4]. Considering this, there might be even fewer cases in which SGs induced by anticancer compounds contribute to drug resistance. Therefore, SIPs cannot be applied to improve the sensitivity of cancer cells to all anticancer drugs, but to a subset of anticancer drugs that induce pro-survival or anti-apoptotic SGs. 

Our study still has certain limitations. First, the length and structure of our current TAT-SIP-C2 (37 aa) and SIP-U1-Antp (33 aa) could be further optimized, which might lead to the identification of the minimal SIPs that retain SG inhibition capacity. A constrained peptide of less than 1200 Daltons might cross the plasma membrane via passive membrane permeability [50,51]. Thus, SIPs less than 10 aa could bypass the use of cell-penetrating peptides, which will expand the application potential of SIPs. If optimized SIPs exceed 1200 Daltons, cell-penetrating peptides could still be skipped by modifying the surface properties of the peptides. These strategies include masking backbone amides to promote passive penetration, presenting a structured pattern of the guanidinium group and amphipathic patterning to promote endocytosis [51]. Second, the induction of SGs is dependent on the cell type and is stress type-specific [24,39,52]. Thus, the SIP-mediated inhibition of SGs might also be cell type and stress type-specific. Although we found that SIPs are able to inhibit AS and sorafenib-induced SGs in HeLa, HepG2, and SH-SY5Y cells, the SG-inhibiting and drug-sensitizing ability of SIPs should be tested with a broader range of cancer cells and SG-inducing anticancer compounds. In addition, further investigation into the mechanisms underlying cell type-specific SG induction and its impact on drug resistance is necessary to better understand the therapeutic potential of SIPs in sensitizing cancer cells to specific anticancer drugs. Last, future studies could investigate the effect of SIPs on SG assembly and drug resistance in vivo, which can be investigated using a tumor xenograft mouse model. 

In summary, our study identified SIPs derived from SG core proteins Caprin1 and USP10. We found that SIPs increase the efficacy of sorafenib via the disruption of sorafenib-induced SGs in HeLa cells. Therefore, the combination of SIPs with SGs-inducing anticancer drugs could sensitize cancer cells, which might provide a novel strategy to alleviate anticancer drug resistance.

## 4. Materials and Methods

### 4.1. Cell Culture and Transfection

Human embryonic kidney epithelial HEK293T cells, human cervical cancer HeLa cells, human hepatocellular carcinoma HepG2 cells, and human neuroblastoma SH-SY5Y cells were grown in Dulbecco-modified Eagle medium (DMEM, BasalMedia, Shanghai, China) supplemented with 10% fetal bovine serum (ExCell Bio, Shanghai, China) and 1% penicillin/streptomycin (Gibco, Gaithersburg, MD, USA) at 37 °C with 5% CO_2_. HeLa cells stably overexpressing GFP-G3BP2 (HeLa/GFP-G3BP2) were grown in DMEM with 10% FBS and 2 μg/mL puromycin [17]. For transfection, cells were seeded in multi-well plates or dishes and transfected with plasmids at approximately 60~70% confluence using PEI (Polyscience, Niles, IL, USA) or lipofectamine 2000 (ThermoFisher, Waltham, MA, USA).

### 4.2. Plasmids 

DNA fragments encoding human Caprin1, G3BP1, G3BP2, and USP10 were amplified from HEK293T cDNA using Q5 high-fidelity DNA polymerase (New England Biolabs, Ipswich, MA, USA), and cloned into pCS2-Flag or pCS2-Myc vectors. Different fragments of Caprin1, G3BP1, USP10, and nsp3 were subcloned into pCS2-GFP-Myc vector. All plasmids were verified by DNA sequencing (Tsingke Biotech, Hangzhou, China). 

### 4.3. SGs Induction and Quantification

To induce oxidative stress, HeLa cells were treated with 0.5 mM sodium arsenite (Sigma-Aldrich, St. Louis, MO, USA) for 45 min. For SGs induced by anticancer drugs, HeLa cells were treated with 10 μM compounds (geldanamycin, vinorelbine and ceritinib) or 50 μM sorafenib for 2 h. G3BP1 or Caprin1 was used as a marker of SGs. For SG counting, the percentage of cells with SGs was analyzed in 100 cells per condition and a minimum of three granules per cell were required for a positive score.

### 4.4. Screen for SG-Inducing Anticancer Compounds

A total of 244 compounds from the anticancer compound library (APExBIO, Shanghai, China) were used to carry out a screen for SG-inducing anticancer compounds. Briefly, HeLa/GFP-G3BP2 cells were seeded to a 48-well plate and cultured to reach 90% confluence. Cells were then treated with 10 μM compound for 2 h and images were acquired with a fluorescence microscope. Compounds that induced SGs with a minimum of three granules per cell and a minimum of 20% SG-positive cells per well were recognized as SG inducers. 

### 4.5. Peptide Synthesis 

Peptides derived from human Caprin1 protein (GenBank #NP_005889.3) and human USP10 protein (GenBank # NP_005144.2) were fused with cell-penetrating peptides TAT or Antp (indicated in italic) and synthesized by GL Biochem (Shanghai, China). The sequences are as follows: TAT-FLAG, NH_2_-*YGRKKRRQRRR*DYKDDDDK-COOH; TAT-SIP-C1 (Caprin1 a.a. 351–390), NH_2_-*YGRKKRRQRRR*DPLVRRQRVQDLMAQMQGPYNFIQDSMLDFENQTLDPAIV-COOH; TAT-SIP-C2 (Caprin1 a.a. 360–385), NH_2_-*YGRKKRRQRRR*QDLMAQMQGPYNFIQDSMLDFENQTL-COOH; SIP-U1-Antp (USP10 a.a. 5–21), NH_2_-SPQYIFGDFSPDEFNQF*RQIKIWFQNRRMKWKK*-COOH.

### 4.6. Immunofluorescence Staining

Immunofluorescence staining was performed as described previously [53]. HeLa cells were fixed in 4% paraformaldehyde for 30 min at 4 ℃ and washed with phosphate buffered saline (PBS) three times. Next, the fixed cells were incubated in blocking solution containing 1% BSA (Sigma-Aldrich, St. Louis, MO, USA) and 1% normal goat serum (Jackson, West Grove, PA, USA) in PBS with 0.1% Triton X-100 for 30 min at room temperature. Cells were then incubated with primary antibodies overnight at 4 °C. The next day, the cells were washed three times with PBS and incubated with secondary antibodies conjugated to Alexa Fluor 488 (ThermoFisher, Waltham, MA, USA) or Alexa Fluor 594 for 1 h at room temperature. Cell nuclei were stained with DAPI (Sigma-Aldrich, St. Louis, MO, USA). Images were acquired with a Zeiss LSM880 confocal microscope. The following antibodies were used: G3BP1 (Santa Cruz Biotech, Dallas, TX, USA, #81940) and Caprin1 (Proteintech, Wuhan, China, #15112-1-AP).

### 4.7. Co-Immunoprecipitation (Co-IP) and Western Blot

Co-IP and Western blot analysis were performed as described previously [16]. Briefly, HEK293T cells were seeded to reach 60~70% confluence in multi-well plates or dishes, and transfected with plasmids for 36 h. Cells were washed with PBS and lysed in lysis buffer containing 50 mM Tris HCl, pH 7.6, 150 mM NaCl, 0.5% NP40, 1 mM EDTA, and protease inhibitor cocktail (APExBIO) at 4 °C for 10 min. Cell lysates were centrifuged twice at 14,000 rpm, 4 °C for 5 min, and supernatant was collected and incubated with an antibody overnight at 4 °C. The next day, 20 μL of protein A/G beads (Smart Lifesciences, Changzhou, China) was added to the supernatant, and the mixture was incubated for 1 h at 4 °C. After washing three times with lysis buffer, the beads were boiled in SDS loading buffer for 5 min, and the supernatant was used for Western blot. The following antibodies were used: Flag (Sigma-Aldrich, St. Louis, MO, USA, #F1804 and #F7425) and Myc (Sigma-Aldrich, St. Louis, MO, USA #M5546).

### 4.8. GST Pull-Down

To obtain glutathione S-transferase (GST)-tagged G3BP1 protein, G3BP1 cDNA was subcloned into bacterial expression vector pGEX-6P-3. Expression plasmid was transformed into *E. coli* strain BL21. A total of 1 mL overnight cultures were inoculated to 100 mL LB medium containing 100 µg of ampicillin/mL and induced for protein expression with 1 mM isopropyl β-D-thiogalactoside (IPTG). After 4 h, the bacterial pellets were collected using centrifugation, resuspended with lysis buffer, and broken using ultrasonication. The broken bacterial liquid was centrifuged at 14,000 rpm at 4 °C for 15 min. The supernatant was incubated with glutathione beads (Smart Lifesciences, Changzhou, China) at 4 °C for 1 h to immobilize bacterially expressed protein. 293T cells were transfected with indicated plasmid for 36 h and harvested for cell lysates. Then, cell lysates were incubated with GST or GST-G3BP1 beads at 4 °C for 1 h. Proteins associated with beads were washed extensively, eluted, and analyzed using Western blot analysis. 

### 4.9. Annexin V/Propidium Iodide (PI) Flow Cytometry

For cell death analysis, HeLa cells were seeded in a 6-well plate at the density of 2 × 10^5^ cells per well and grown for 24 h. After cells reached 90% confluence, cells were pretreated with 200 μM peptides for 2 h, and treated with 50 μM sorafenib for an additional 2 h. Then, cells were digested with trypsin, washed once with cold PBS, and resuspended in 1 × binding buffer. The Annexin V/PI apoptosis detection kit (Sangon Biotech, Shanghai, China) was employed for analysis of cell death according to the manufacturer’s instructions. Double negative cells (Annexin V^−^PI^−^) were counted as living cells, and single positive (Annexin V^+^PI^−^ or Annexin V^−^PI^+^) and double positive (Annexin V^+^PI^+^) cells were combined to count as dead cells. 

### 4.10. Analysis of Protein–Protein Interaction Network and Intrinsically Disordered Region (IDR)

The G3BP1 protein–protein interaction network was obtained from STRING (https://cn.string-db.org, accessed on 16 February 2024), which is a database of known and predicted protein–protein interactions [54]. The analysis of human G3BP1 network was performed following the website instructions. In the basic setting, a physical subnetwork was selected as the network type while all other parameters were set by default. Therefore, only direct or physical interactions were included whereas indirect or functional associations were excluded. IDRs of Caprin1, G3BP1, and USP10 were predicted by PONDR (http://www.pondr.com/, accessed on 16 February 2024), an online predictor of natural disordered regions [55]. 

### 4.11. Statistical Analysis

Statistical analyses were performed with GraphPad Prism. All results are expressed as the mean ± SD of at least three independent experiments. An unpaired Student’s *t*-test was used to compare the means between the groups. In all tests, *p* > 0.05 was considered not statistically significant (n.s.), and *p* ≤ 0.05 was considered statistically significant (* *p* ≤ 0.05).

## Data Availability

All generated and analyzed data used to support the findings of this study are included within the article.
